# Correlation of CA-125 serum level and clinico-pathological characteristic of patients with endometriosis 

**Published:** 2016-11

**Authors:** Mojgan Karimi-Zarchi, Najmeh Dehshiri-Zadeh, Leili Sekhavat, Fahime Nosouhi

**Affiliations:** 1 *Department of Obstetrics and Gynecology, School of Medicine, Shahid Sadoughi University of Medical Sciences, Yazd, Iran.*; 2 *Recurrent Abortion Research Center, Research and Clinical Center for Infertility, Shahid Sadoughi University of Medical Sciences, Yazd, Iran.*; 3 *Faculty of Pharmacy, School of Medicine, Shahid Sadoughi University of Medical Sciences, Yazd, Iran.*

**Keywords:** *CA125*, *Endometriosis*, *Pelvic pain*, *Stage*, *Mass size*, *Adhesion score*

## Abstract

**Background:**

Cancer antigen 125 (CA-125) is a glycoprotein biomarker that is used in women with pelvic masses such as endometriosis and maybe is useful in practice of patients suspicious to endometriosis.

**Objective:**

The aim of this study was to evaluate the association between preoperative serum CA-125 levels and clinic pathological characteristic in women with endometriosis, and find out the best serum CA-125 levels cut-off in pre and post menopause women.

**Materials and Methods:**

Serum CA-125 levels in 87 women aged 21-54 years suspected to endometriosis with pelvic pain, dysmenorrhea, or dyspareunia were measured preoperatively. Also the association between clinic pathological characteristic and serum CA-125 level were analyzed.

**Results:**

The mean age of women was 32.22±6.91. The mean serum CA-125 level was 49.93±4.30 U/mL. There was a significant correlation between the endometriosis stage, lesion size, adhesion score and preoperative CA-125 plasma concentration. However, we did not found significant differences in age, marital status, patient’s complaints, and pelvic pain associated to Ca125 serum level. The suggested preoperative serum cut-off levels in premenopausal and postmenopausal patients were 37 U/ml and 35 U/ml, respectively.

**Conclusion:**

According to the results, preoperative serum CA-125 is an important predictor for patients with endometriosis and it should be taken into consideration when surgical management is suspected, especially if stage of disease, lesion size and adhesion score are undertaken.

## Introduction

Endometriosis is an estrogen dependent gynecologic disease with lasting implications for many women's fertility, somatic health, and overall quality of life. Endometriosis is seen in approximately 10-15% of adult women aged 25-35 years old (1). This disorder occurs when the endometrial tissue (cells that line the uterus) grows in other areas of the body. Endometriosis causes varying degrees of painful symptoms and infertility in infected individuals (2). The standard treatment of endometriosis in women who have suitable number of babies is surgery, including hysterectomy, bilateral salpingo-oophorectomy (3). Cancer antigen 125 (CA-125) is a glycoprotein biomarker that is used in women with pelvic masses such as endometriosis and maybe is useful in practice of patients suspicious to endometriosis (3).

It serves as an ovarian cancer biomarker, especially for monitoring of ovarian cancer therapy and early recurrences (4, 5). CA-125 levels have been found to be significantly higher in women with moderate or severe endometriosis (6). It was reported that preoperative serum CA-125 level could be used as an important predictor for patients with endometrial conditions. Most common benign gynecological conditions associated with high serum CA-125 are ovarian endometrioma and deeply infiltrating endometriosis (7). Due to endometriosis similarity and ovarian malignancy in radiological markers and CA-125 serum level and the effect of surgery on ovarian reserve in women with endometriosis, our main goal was to evaluate the correlation between CA-125 serum level and the clinic pathological characteristics in women referred to our center. 

Our specific goal was to determine the best CA-125 cut-off level in pre- and postmenopausal women with endometriosis. It seems preoperative serum CA-125 levels can be used as a guide to perform aggressive surgical staging with regards to initial treatment modalities as well as to predict prognosis (9, 10). There are so many clues that elevated preoperative serum CA-125 levels were strongly correlated with advanced-stage disease, higher grade, deep myometrial invasion, lymph node metastases, presence of extra-uterine spread of the tumor and poor prognosis (11, 12). Hetergenecity of adnexal mass is one of the most common endometriosis features. Also higher level of CA125 is common in women with endometriosis. Both of these points are important for patient selection for surgery suspected to malignancy. 

In older and menopausal women these characteristics help to surgeons to do surgery as soon as possible.

## Materials and methods

This cross-sectional study was conducted on 87 women suspected endometriosis with pelvic pain, dysmenorrhea, or dyspareunia referred to Obstetrics and Gynecology department of Shahid Sadoughi Hospital, between March 2013 and July 2014 for laparoscopy or laparotomy. The study was approved by the Shahid Sadoughi Medical Science University Ethics Committee. A written informed consent was obtained from all patients. The subjects were free to participate in the study.

Inclusion criteria were women with pelvic pain, dysmenorrhea, or dyspareunia, suspected endometriosis, and aged 21-54 years. As a matter of fact women within 45-54 years who radiological findings showed endometriosis were entered to the study. Blood samples were preoperatively obtained from all CA-125 participants levels measurement up to 2 days before surgery. Serum CA-125 levels were determined by sandwich type enzyme-linked immunosorbent assay (ELISA) kit monoclonal (Roche, Germany). 

The upper normal value of serum CA-125 levels was considered 35 U/mL. All women with history of medical or surgical treatments for endometriosis in the past three months, previous history of pelvic surgery, history of pelvic inflammatory disease, and a suspected or certain diagnosis of malignancy in premenopausal women, women with chronic pelvic pain resulting only from musculoskeletal, infectious, neurologic, gastrointestinal or psychiatric causes, refusal to participate in the study pregnancy and lactation were excluded. 

Endometriosis was diagnosed according to the clinical symptoms and sonographic findings and was staged according to the classification of the American Society for Reproductive Medicine, as revised in 1996 (23). Preoperative pain was assessed by visual analogue scale and analgesic usage (VAS). All subjects completed a preoperative questionnaire concerning on menstrual history, marital status, age, body mass index, professional activity, medical and surgical history and characteristics of pain symptoms. Women completed the questionnaire alone, without any assistance of others. Women were questioned for pain symptoms within three months before the surgery. Pelvic pain was assessed by a visual analogue scale (VAS; 0 absence of pain, 10 unbearable pain). VAS is a suitable and well-proved tool for the measurement of pelvic pain associated with endometriosis.

Surgical finding such as the adhesions severity, the extent and location of endometriosis and stage of endometriosis were recorded in a checklist by gynecologic laparoscopy specialist. Stage of endometriosis was assigned according to revised-American Fertility Society (r-AFS) stages I-IV and score of adhesion were defined according to the revised the modified American Fertility Society mAFS scoring method for adhesions (3).

**Statistical analysis**

Data were analyzed using SPSS statistical software (SPSS, Chicago, IL, USA). The data on serum CA-125 levels in the patients did not have a normal distribution. Therefore, a nonparametric test was used to evaluate its relation with clinic pathological characteristic. The levels of serum CA-125 in different group were analyzed using Mann-Whitney U test and a Pearson ^2^ test. Receiver operating characteristic (ROC) curve analysis was used to find a cut-off level of CA-125 in serum with optimal diagnostic sensitivity and specificity. For all analyses, values of p<0.05 were considered significant.

## Results

The mean age of participants was 32.22±6.91 (ranged, 21-54 years). Out of 87 women, 12 cases were in menopausal age referred with pain and underwent surgery for suspected cancer. However, the surgery results shown that they were suffering from endometriosis. The mean serum CA-125 level of women with endometriosis was 49.93±4.30 U/mL (range, 2-191 U/mL). 

Association between preoperative serum CA-125 and clinicopathological characteristics are listed in Table I. The elevated CA-125 level was significantly associated with clinicopathological parameters, including stage of disease (p≤0.001), adhesion score (p=0.04), and lesion size (p=0.02) and preoperative CA-125. There was no significant differences in age (p=0.76), marital status (p=0.85), patient’s complaints (p=0.20), and pelvic pain score (p=0.70) associated to presurgical CA125 serum level. The best cut-off level based menopausal or postmenopusal status-, the CA-125 serum levels was 37 U/ml for premenopause patients with 57% sensitivity, and 50% specificity, and 35 U/ml for postmenopause patients with 70% sensitivity, and 59% specificity.

**Table I:** Association between preoperative serum CA-125 and clinicopathological characteristics

**Characteristic**		**Serum CA-125 level**	**p-value**
Age (yrs)			
	≤40	55.22 ± 13.27	0.76
	>40	52.21 ± 12.59	
Marital status			
	Single	35.27 ± 12.47	0.85
	Married	40.81 ± 4.59	
Stage of disease			
	1	29.38 ± 5.81	≤0.001
	2	53.20 ± 6.64	
	3	56.12 ± 6.88	
	4	49.91 ± 7.51	
Adhesion score			
	1-2	29.38 ± 5.81	0.04
	3-4	53.20 ± 6.64	
	5-6	56.12 ± 6.88	
Complaint			
	Pelvic pain	47.67 ± 5.20	0.20
	Infertility	45.12 ± 1.98	
	Infertility + Pain	61.90 ± 20.7	
AUB		32.40 ± 8.30	
AUB + Pain		60.65 ± 8.99	
Lesion size (Cm)			
	≤4.5	49.91 ± 7.51	0.02
	5-11	49.69 ± 5.32	
VAS			
	2	52.80 ± 14.55	0.70
	2-5	43.93 ± 4.65	
	6-9	55.43 ± 8.46	

Data presented as mean±SE.

AUB: Abnormal uterine bleeding

VAS: Visual analogue scale

## Discussion

The mean serum CA-125 level of women with endometriosis was 49.93±4.30 U/mL (range, 2-191 U/mL) in this study. Amara and colleagues found that CA-125 serum levels were higher in endometriosis patients when compared to control group during both periods of menstrual cycle (13). Also, Emin and co-workers showed that CA-125 frequently elevated in patients with endometriosis (14).

In the current study, there was no significant difference in terms of age, marital status, and complaints associated to presurgical CA125 level. Our data showed that serum CA-125 level in patients with pelvic pain, infertility, and infertility with pain has been increased. However, there was no significant association between serum CA-125 level and the complaints that referred to our clinic. Ramos *et al* reported a significant increased CA-125 serum level in the infertile endometriosis women than fertile and never tried ones (15). It was reported a correlation between CA-125 levels and the proliferative activity of epithelial cells in the endometriosis lesions, because the disease is an inflammatory process associated with a change in immune cell functions (16*, *17*)*.

In this study, we have found a strong association between preoperatively elevated CA-125 levels and advanced stage of disease. Our finding runs in agreement with previous studies in which advanced endometriosis was associated with high level of CA-125 in the serum (18). Amaral *et al* reported that women with more advanced degrees of endometriosis showed higher CA-125 levels in both serum and peritoneal fluid (13). It seems the elevation of CA-125 in women with endometriosis is because of its higher concentration in ectopic than in entopic endometrium (13, 18). Moreover, it may be due to inflammatory reactions, which alters the endothelial permeability leading the marker to reach the circulation (13). Therefore, preoperative serum CA-125 assessment should be considered in all patients with suspected advanced disease stage or presence of unfavorable histology in endometrial biopsy as an adjunct to the stage prediction of disease and subsequent patient management. Moreover, serum CA-125 levels were notably higher in women with larger adhesions to the peritoneum, fallopian tube, ovary, omentum, colon, and cul-de-sac, or with ruptured endometrioma (18). 

It was also found that women with endometriosis with preoperative CA-125 more than 65 IU/mL are at high risk for intense pelvic adhesions (18). Also Franssen *et al* reported that menstruation and adhesions appears to be the main factors affecting pretreatment serum CA-125 level in patients with endometriosis (15). Garzetti and co-workers found that serum CA-125 concentration is directly correlated to the adhesion score and peritoneal (19). Besides, it was reported that CA-125 increases the invasiveness of a benign endometriosis cell line and affects cell adhesion *in vitro* (19).

The normal CA-125 level in postmenopausal women is <15 U/mL, which is significantly lower than in premenopausal women (20, 21). In a meta-analysis, Bedaiwy and Falcone reviewed the Medline database for studies about CA-125 performance in testing endometriosis. Most studies included in the meta-analysis accepted the value of 35 U/ml as a cut-off level for CA-125 serum concentration (22). In the current study, the preoperative serum cut-off level of CA-125 (37 U/ml) in premenopausal patients was slightly higher than postmenopausal patients (35 U/ml). 

The value is normal range for CA-125 concentration in postmenopausal patients. The sensitivity and specificity were 57%, 50% for premenopausal patients and 70% and 59% in the postmenopausal patients, respectively. The results of the Bedaiwy and Falcone meta-analysis showed that sensitivity of serum CA-125 was varied in a wide range from 24-94% (22). In a study, Szubert *et al* reported that the sensitivity of serum concentration of CA-125 in the diagnosis of disease was 68% reaching up to 91.67% for the diagnosis of advanced stages of endometriosis. Considerably, they have reported, the cut-off value in serum suggesting endometriosis with 68% sensitivity is 11 U/ml (7). Therefore, the best cut-off level was different for premenopausal or ≤50 years and menopausal or >50 years patients. 

## Conclusion

Preoperative serum CA-125 is an important predictor for patients with endometriosis and it should be taken into consideration when surgical management is suspected, especially if the stage of disease, lesion size and adhesion score are undertaken.

## Note

***This article extracted from M.D. thesis. (Najmeh Dehshiri-Zadeh)***

## References

[1] Hirsch M, Duffy JM, Kusznir JO, Davis CJ, Plana MN, Khan KS (2016). International Collaboration to Harmonize Outcomes and Measures for Endometriosis Variation in outcome reporting in endometriosis trials: a systematic review. Am J Obstet Gynecol.

[2] Barbieri RL, Niloff JM, Bast RC Jr, Scaetzl E, Kistner RW, Knapp RC (1986). Elevated serum concentrations of CA-125 in patients with advanced endometriosis. Fertil Steril.

[3] Aflatoonian A, Rahmani E, Rahsepar M (2013). Assessing the efficacy of aspiration and ethanol injection in recurrent endometrioma before IVF cycle: A randomized Clinical Trial. Iran J Reprod Med.

[4] Jiang T, Huang L, Zhang S (2015). Preoperative serum CA125: a useful marker for surgical management of endometrial cancer. BMC Cancer.

[5] Pittaway DE, Rondinone D, Miller KA, Barnes K (1995). Clinical evaluation of CA-125 concentrations as a prognostic factor for pregnancy in infertile women with surgically treated endometriosis. Fertil Steril.

[6] Koninckx PR (1994). Is mild endometriosis a condition occurring intermittently in all women?. Hum Reprod.

[7] Szubert M, Suzin J, Wierzbowski T, Kowalczyk-Amico K (2012). CA-125 concentration in serum and peritoneal fluid in patients with endometriosis- preliminary results. Arch Med Sci.

[8] Shiau CS, Chang MY, Chiang CH, Hsieh CC, Hsieh TT (2003). Ovarian endometrioma associated with very high serum CA‐125 levels. Chang Gung Med J.

[9] Chen YL, Huang CY, Chien TY, Huang SH, Wu CJ, Ho CM (2011). Value of pre-operative serum CA125 level for prediction of prognosis in patients with endometrial cancer. Aust N Z J Obstet Gynaecol.

[10] Kim HS, Park CY, Lee JM et al (2010). Evaluation of serum CA-125 levels for preoperative counseling in endometrioid endometrial cancer: a multi-center study. Gynecol Oncol.

[11] Powell JL, Hill KA, Shiro BC, Diehl SJ, Gajewski WH (2005). Preoperative serum CA-125 levels in treating endometrial cancer. J Reprod Med.

[12] Chung HH, Kim JW, Park NH, Song YS, Kang SB, Lee HP (2006). Use of preoperative serum CA-125 levels for prediction of lymph node metastasis and prognosis in endometrial cancer. Acta Obstet Gynecol Scand.

[13] Amaral VF, Ferriani RA, Sá MF, Nogueira AA, Rosa e Silva JC, Rosa e Silva AC (2006). Positive correlation between serum and peritoneal fluid CA-125 levels in women with pelvic endometriosis. Sao Paulo Med J.

[14] Emin U, Tayfun G, Cantekin I, Ozlem UB, Umit B, Leyla M (2009). Tumor markers in mature cystic teratomas of the ovary. Arch Gynecol Obstet.

[15] Ramos I, Podgaec S, Abrão M, Oliveira R, Baracat E (2012). Evaluation of CA-125 and soluble CD-23 in patients with pelvic endometriosis: a case-control study. Revista da Associação Médica Brasileira (English Edition).

[16] Cho S, Cho H, Nam A, Kim HY, Choi YS, Park KH (2008). Neutrophil-to-lymphocyte ratio as an adjunct to CA-125 for the diagnosis of endometriosis. Fertil Steril.

[17] Abrão MS, Gonçalves MOC, Dias Jr JA, Podgaec S, Chamie LP, Blasbalg R (2007). Comparison between clinical examination, transvaginal sonography and magnetic resonance imaging for the diagnosis of deep endometriosis. Hum Reprod.

[18] Cheng YM, Wang ST, Chou CY (2002). Serum CA-125 in preoperative patients at high risk for endometriosis. Obstet Gynecol.

[19] Garzetti GG, Ciavattini A, Tranquilli AL, Arduini D, Romanini C (1994). Serum CA-125 concentration in endometriosis patients: role of pelvic and peritoneal irritation. Gynecol Endocrinol.

[20] Kukura V, Zaninovic I, Hrdina B (1990). Concentrations of CA 125 tumor marker in endometrial carcinoma. Gynecol Oncol.

[21] Takami M, Sakamoto H, Ohtani K, Takami T, Satoh K (1997). An evaluation of CA 125 levels in 291 normal postmenopausal and 20 endometrial adenocarcinoma-bearing women before and after surgery. Cancer Lett.

[22] Bedaiwy MA, Falcone T (2004). Laboratory testing for endometriosis. Clin Chim Acta.

[23] Karimi-Zarchi M, Baghdadabad A, Baghdadabad MR, Zahir ShT, Abadi RD, Teimoori S (2014). Evaluation of serum CA 125 level and its relation to surgical, histopathologic and ultrasonographic findings in patients with pelvic mass. Eur J Gynaecol Oncol.

